# Disconnect between the effects of serelaxin on renal function and outcome in acute heart failure

**DOI:** 10.1007/s00392-022-02144-6

**Published:** 2023-01-19

**Authors:** I. E. Beldhuis, J. M. ter Maaten, S. M. Figarska, K. Damman, P. S. Pang, B. Greenberg, B. A. Davison, G. Cotter, T. Severin, C. Gimpelewicz, G. M. Felker, G. Filippatos, J. R. Teerlink, M. Metra, A. A. Voors

**Affiliations:** 1 Department of Cardiology, University Medical Center Groningen, University of Groningen, Hanzeplein 1, 9713 GZ Groningen, The Netherlands; 2 Department of Emergency Medicine, Indiana University, Indianapolis, IN USA; 3 Sulpizio Family Cardiovascular Center, University of California San Diego Health, La Jolla, CA USA; 4 Momentum Research and Inserm U942 MASCOT, Paris, France; 5 Novartis Pharma, Basel, Switzerland; 6 Duke University School of Medicine and Duke Clinical Research Institute, Durham, NC USA; 7 Department of Cardiology, Athens University Hospital Attikon, National and Kapodistrian University of Athens, School of Medicine, Athens, Greece; 8 Section of Cardiology, San Francisco Veterans Affairs Medical Center and School of Medicine, University of California, San Francisco, CA USA; 9 Cardiology, Department of Medical and Surgical Specialties, Radiologic Sciences, and Public Health, University of Brescia, Brescia, Italy

**Keywords:** Acute heart failure, Renal function change, Serelaxin, Mediation analysis

## Abstract

**Background:**

We aimed to study whether improvement in renal function by serelaxin in patients who were hospitalized for acute heart failure (HF) might explain any potential effect on clinical outcomes.

**Methods:**

We included 6318 patients from the RELAXin in AHF-2 (RELAX-AHF2) study. Improvement in renal function was defined as a decrease in serum creatinine of ≥ 0.3 mg/dL and ≥ 25%, or increase in estimated glomerular filtration rate of ≥ 25% between baseline and day 2. Worsening renal function (WRF) was defined as the reverse. We performed causal mediation analyses regarding 180-day all-cause mortality (ACM), cardiovascular death (CVD), and hospitalization for HF/renal failure.

**Results:**

Improvement in renal function was more frequently observed with serelaxin when compared with placebo [OR 1.88 (95% CI 1.64–2.15, *p* < 0.0001)], but was not associated with subsequent clinical outcomes. WRF occurred less frequent with serelaxin [OR 0.70 (95% CI 0.60–0.83, *p* < 0.0001)] and was associated with increased risk of ACM, worsening HF and the composite of CVD and HF or renal failure hospitalization. Improvement in renal function did not mediate the treatment effect of serelaxin [CVD HR 1.01 (0.99–1.04), ACM HR 1.01 (0.99–1.03), HF/renal failure hospitalization HR 0.99 (0.97–1.00)].

**Conclusions:**

Despite the significant improvement in renal function by serelaxin in patients with acute HF, the potential beneficial treatment effect was not mediated by improvement in renal function. These data suggest that improvement in renal function might not be a suitable surrogate marker for potential treatment efficacy in future studies with novel relaxin agents in acute HF.

**Graphical abstract:**

Central illustration. Conceptual model explaining mediation analysis; treatment efficacy of heart failure therapies mediated by renal function.

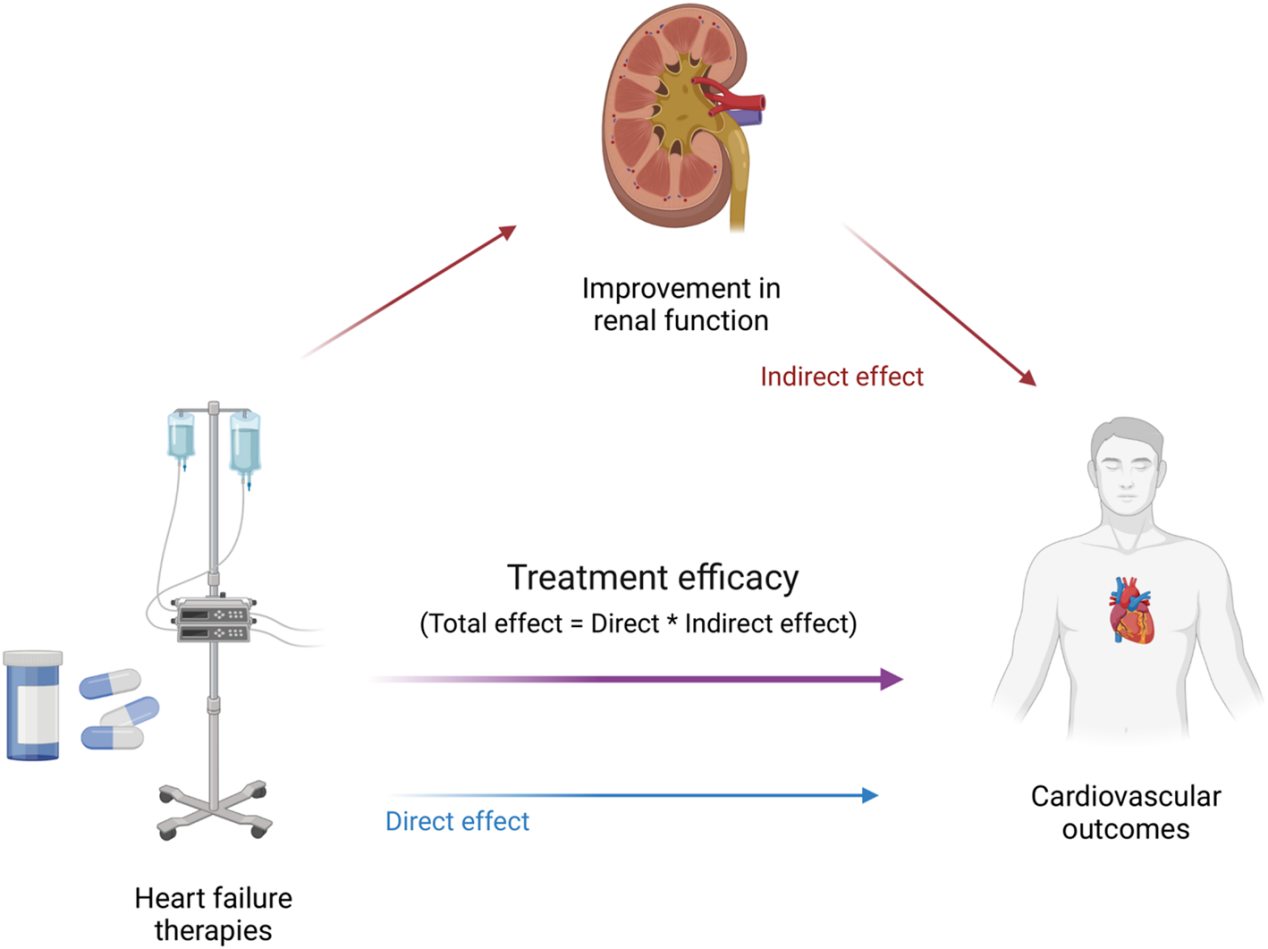

**Supplementary Information:**

The online version contains supplementary material available at 10.1007/s00392-022-02144-6.

## Introduction

In patients with heart failure (HF), chronic kidney disease (CKD) is a frequently observed co-morbidity, with prevalence up to 20–70%, and is associated with increased risk of cardiovascular (CV) outcomes [1–3]. Although worsening renal function (WRF) is generally thought to be deleterious, changes in renal function during a heart failure hospital admission have been variably associated with clinical outcomes. In some studies, WRF was contrastingly associated with better clinical outcomes, while improvement in renal function has been associated with worse outcomes [4, 5].

Serelaxin is a recombinant form of human relaxin-2, which as a vasoactive peptide hormone regulates cardiovascular and renal adaptations in pregnancy, including increased arterial compliance, cardiac output and renal blood flow, potentially relevant to the treatment of acute HF [6]. Although preliminary beneficial effects of serelaxin on cardiovascular mortality in the RELAXin in Acute Heart Failure (RELAX-AHF) trial could not be confirmed in the much larger RELAX-AHF2 trial, in both trials, serelaxin consistently showed beneficial effects on renal function [7–9].

Currently, several novel relaxin agents are being developed for the treatment of patients with HF. In phase II trials, improvement in renal function is often considered a surrogate endpoint for efficacy in potential phase III trials. It is generally assumed that drugs that improve renal function might have beneficial effects on mortality and HF hospitalizations as well. However, evidence for this assumption is still lacking. Therefore, we aimed to establish to what extent the effects of serelaxin on cardiovascular mortality at 180 days could be mediated by improvement in renal function.

## Methods

### Patient population

The derivation cohort of this study was the RELAX-AHF-2 trial, a multinational, randomized, placebo-controlled, double-blind, phase-3 study evaluating the effects of serelaxin infusion versus placebo on clinical outcomes in patients with acute HF. The rationale and design of the study have been published previously [10, 11]. In brief, the RELAX-AHF-2 trial included 6545 patients ≥ 18 years of age with acute HF, mild-to-moderate renal insufficiency (eGFR of 25–75 mL/min/1.73 m^2^), increased brain natriuretic peptide (BNP) or N-terminal prohormone of BNP, with dyspnea, congestion on chest radiograph and systolic blood pressure > 125 mmHg. Patients were randomized within 16 h after hospital admission in a 1:1 ratio to either serelaxin 30 µg/kg per day continuously in the first 48 h or placebo. All patients provided informed consent, and protocol was reviewed and approved by the applicable institutional review boards or ethics committees prior to study start. We have included patients who had serum creatinine measurements available at baseline and day 2, resulting in a total population of 6318 patients.

#### Sensitivity analyses

Sensitivity analyses were performed in the earlier RELAX-AHF trial. The RELAX-AHF trial, with similar key inclusion and exclusion criteria and therefore similar patient population, included 1161 eligible patients with an eGFR between 30 and 75 mL/min/1.73 m^2^. The rationale and design of the study have been published previously [11]. All patients provided informed consent, and protocol was reviewed and approved by the applicable institutional review boards or ethics committees prior to study start.

### Study outcomes

The main clinical endpoint of interest was 180-day cardiovascular mortality, which was the co-primary endpoint of the RELAX-AHF2 trial. Other endpoints of interest included all-cause mortality at 180 days and HF or renal failure re-hospitalization at 180 days.

For improvement in renal function, a literature-based definition was used, defining it as an absolute decrease in serum creatinine ≥ 0.3 mg/dL and a relative decrease in serum creatinine of ≥ 25% or a relative increase in eGFR (CKD-EPI formula) of ≥ 25%, between baseline and day 2. Day 2 was selected because it marked the end of the Serelaxin infusion period [1, 3–5]. WRF was defined as an absolute decrease in serum creatinine ≥ 0.3 mg/dL and a relative increase in serum creatinine of ≥ 25% or a relative decrease in eGFR (CKD-EPI formula) of ≥ 25%, between baseline and day 2 (48 h). Continuous change in renal function was defined as change in eGFR (CKD-EPI formula) from baseline to day 2 (48 h).

### Statistical analysis

The population was divided into groups according to the occurrence of improvement in renal function and/or treatment allocation at baseline, for both trials separately. Baseline characteristics are presented by improvement in renal function categories as mean ± SD, median (interquartile range [IQR]), or numbers (percentages) as appropriate. ANOVA test was used to compare groups for continuous variables normally distributed, Kruskal–Wallis test for non-normally distributed continuous variables, and Chi-square tests or Fisher’s exact test for categorical variables.

Univariable and multivariable predictors at baseline for improvement in renal function were obtained using logistic regression models. Univariable predictors were transferred to the multivariable model if *p* < 0.10. For multivariable modelling, backward elimination (*p* > 0.05) was performed until all variables remained significant (*p* < 0.05). Cox regression models were used to evaluate association with outcome. Multivariable adjustment was performed according to previously published models [12]. Estimates are presented as hazard ratios (HRs) with 95% confidence intervals (CIs).

#### Mediation analysis

Mediation analysis model for causal effects was used with cardiovascular or all-cause death at day 180 as time-to-event outcome, and using improvement in renal function between baseline and day 2 as a binary mediator. For the binary mediator analysis, a multivariable logistic regression model was performed with improvement in renal function as outcome variable. Variables included in the model were selected using backward elimination strategy. Regarding the survival analysis with the time-to-event outcome, Cox proportional hazard models were used.

Two-tailed *p* values < 0.05 were considered statistically significant. All statistical analyses were performed using R statistical software version 4.1.0 (R Foundation for Statistical Computing, Vienna, Austria).

## Results

Baseline characteristics of the 1089 (17%) patients who experienced improvement in renal function between baseline and day 2 and 5229 (83%) patients without improvement in renal function from the RELAX-AHF2 cohort are presented in Table 1. Patients who experienced improvement in renal function were younger, were more frequently male, had lower baseline systolic blood pressure, were lower ejection fraction, were more frequently treated with betablockers and MRA at admission, had higher baseline creatinine concentration and corresponding lower baseline eGFR (Tables 1, 2, Supplementary Table 1). Baseline characteristics between those with and without improvement in renal function were similar in RELAX-AHF (Supplementary Table 2, Supplementary Table 3).

**Table 1:** Baseline characteristics for patients who did and did not experience improvement in renal function between baseline and day 2

RELAX-AHF2 *N* = 6318	No IRF	IRF	*p* value
	*N* = 5229 (83%)	*N* = 1089 (17%)	
Demographics			
Age, years	73 (11)	71 (12)	< 0.001
Female	2136 (41)	407 (37)	0.04
White race	4819 (92)	1004 (92)	0.66
Body mass index, kg/m^2^	30 (6)	30 (7)	0.15
LVEF, %	40 (14)	39 (14)	0.02
HFrEF	2495 (51)	589 (58)	< 0.001
NYHA functional class			0.44
I	170 (5)	33 (4)	
II	1481 (39)	314 (39)	
III	1745 (46)	366 (45)	
IV	394 (10)	100 (12)	
Previous HFH	2664 (55)	571 (56)	0.42
Ischemic etiology	2096 (54)	434 (53)	0.37
Systolic blood pressure, mmHg	142 (15)	139 (14)	< 0.001
Heart rate, beats/min	81 (16)	82 (17)	0.08
Biomarkers			
Creatinine, mg/dL	1.34 (0.39)	1.45 (0.36)	< 0.001
Baseline eGFR CKD-EPI, mL/min/1.73 m^2^	51.0 (15.2)	47.1 (13.8)	< 0.001
Day 2 eGFR CKD-EPI, mL/min/1.73 m^2^	48.9 (16.8)	67.1 (19.2)	< 0.001
Potassium, mmol/L	4.3 (0.6)	4.3 (0.6)	0.12
Hemoglobin, g/L	126 (19.5)	129 (19.9)	< 0.001
BUN, mg/dL	23.9 [18.5; 31.7]	25.5 [19.6; 34.5]	< 0.001
AST, IU/L	26.0 [20.0; 35.0]	29.4 [21.0; 42.6]	< 0.001
NT-proBNP, ng/L	5231 [2990; 9763]	5337 [2862; 9429]	0.73
Medical therapy			
ACEi/ARB	3433 (69)	710 (70)	0.69
Beta-blocker	3704 (75)	792 (78)	0.03
MRA	1468 (30)	349 (34)	0.003
Treatment			< 0.001
Placebo	2743 (53)	405 (37)	
Serelaxin, 30 µg/kg/day	2456 (47)	681 (63)	

Values are mean ± SD, frequency (percentage), or median (interquartile range)

*ACEi* angiotensin-converting enzyme inhibitor, *ARB* angiotensin receptor blocker, *AST* aspartate aminotransferase, *BUN* blood urea nitrogen, *CKD-EPI* chronic kidney disease epidemiology collaboration, *eGFR* estimated glomerular filtration rate, *HFH* heart failure hospitalization, *IRF* improvement in renal function, *LVEF* left ventricular ejection fraction, MRA mineralocorticoid receptor antagonist, *NT-proBNP* N-terminal prohormone brain natriuretic peptide, *NYHA* New York Heart Association

### Effects of serelaxin on improvement in renal function

In RELAX-AHF2, improvement in renal function was observed in 681 (22%) patients treated with serelaxin compared with 405 (13%) patients receiving placebo (*p* < 0.0001). Consequently, treatment with serelaxin was associated with 1.88 times higher odds of improvement in renal function (OR 1.88; 95% CI 1.64–2.15, *p* < 0.0001), when compared to patients receiving placebo.

**Table 2:** Multivariable predictors at baseline of improvement in renal function

RELAX-AHF-2		
Variable	Odds ratio (95% CI)	*p* value
Age	0.98 (0.97–0.99)	< 0.0001
SBP	0.99 (0.98–0.99)	< 0.0001
Creatinine, mg/dL	2.39 (1.97–2.89)	< 0.0001
ALT, IU/L	1.00 (1.00–1.01)	< 0.0001
Hematocrit	5.71 (1.56–20.93)	0.008
Serelaxin treatment	1.90 (1.65–2.20)	< 0.0001
Male sex	0.78 (0.66–0.92)	0.002
Diabetes status	0.80 (0.69–0.93)	0.003

*CI* confidence interval, other abbreviations as in Table 1

Development of worsening renal function was less frequently observed with serelaxin treatment, compared with placebo [265 (8%) patients vs. 365 (12%); OR 0.70; 95% CI 0.60–0.83, *p* < 0.0001].

Similar results were observed in the sensitivity analyses performed in the RELAX-AHF cohort (Supplementary Table 4).

### Association between change in renal function and clinical outcomes

In the overall RELAX-AHF2 population, improvement in renal function between baseline and day 2 was not associated with the risk of the primary and secondary outcomes (Table 3). In the serelaxin group, improvement in renal function was not associated with all-cause mortality or other CV outcomes. In the placebo group, improvement in renal function was associated with higher risk of CV death [HR 1.47 (95% CI 1.04–2.09), *p* = 0.03, *p*-interaction = 0.04] (Tables 3, 4).

**Table 3:** Improvement in renal function/worsening renal function and relation to outcome in cox regression analysis

RELAX-AHF-2	All-cause mortality 180 days^a^		CV death 180 days		Worsening HF 5 days		HF/renal failure hospitalization 180 days		CV death or HF/renal failure hospitalization 180 days	
Univariable	Hazard ratio (95% CI)	*p* value	Hazard ratio (95% CI)	*p* value	Hazard ratio (95% CI)	*p* value	Hazard ratio (95% CI)	*p* value	Hazard ratio (95% CI)	*p* value
IRF	1.15 (0.96–1.39)	0.14	1.23 (1.00–1.52)	0.05	0.84 (0.65–1.09)	0.20	0.92 (0.78–1.07)	0.27	0.98 (0.86–1.12)	0.79
WRF	1.08 (0.85–1.37)	0.51	1.04 (0.79–1.38)	0.77	1.64 (1.27–2.23)	0.0002	1.07 (0.89–1.28)	0.50	1.14 (0.97–1.33)	0.12
Age/sex										
IRF	1.24 (1.02–1.45)	0.03	1.30 (1.05–1.60)	0.02	0.86 (0.66–1.12)	0.27	0.92 (0.79–1.08)	0.32	1.00 (0.88–1.15)	0.96
WRF	1.04 (0.82–132)	0.75	1.01 (0.76–1.33)	0.97	1.64 (1.26–2.21)	0.0002	1.07 (0.88–1.28)	0.51	1.13 (0.96–1.32)	0.15
Full model^a^										
IRF	0.99 (0.79–1.25)	0.92	1.09 (0.85–1.40)	0.50	0.82 (0.6–1.12)	0.21	0.87 (0.72–1.04)	0.13	0.91 (0.77–1.06)	0.22
WRF	1.39 (1.06–1.82)	0.02	1.31 (0.96–1.79)	0.09	1.97 (1.46–2.67)	< 0.0001	1.12 (0.90–1.40)	0.31	1.24 (1.03–1.49)	0.02
Treatment interaction										
IRF		0.03		0.04		0.56		0.95		0.38
WRF		0.51		0.15		0.69		0.45		0.10

^a^CV death/All-cause mortality; creatinine (µmol/L); hemoglobin (g/L); sodium (mmol/L); blood urea nitrogen (mg/dL); cerebrovascular accident; asthma/bronchitis/COPD; peripheral arterial occlusive disease; respiration rate (breaths/min); systolic blood pressure (mmHg); body mass index (kg/m^2^); edema; IV loop diuretics (total dose in furosemide units) at baseline; known history of diabetes mellitus; prior heart failure hospitalization; actual study treatment; composite of NT-proBNP or BNP *Z*-score; sex; age (years) and LVEF per 5. Worsening HF, HF/renal failure hospitalization, CV death/HF/renal failure hospitalization: creatinine (µmol/L); hemoglobin (g/L); sodium (mmol/L); blood urea nitrogen (mg/dL); cerebrovascular accident; depression; asthma/bronchitis/COPD; atrial fibrillation/flutter; peripheral arterial occlusive disease; pulse (beats/min); respiration rate (breaths/min); systolic blood pressure (mmHg); edema; IV loop diuretics (total dose in furosemide units) at baseline; known history of diabetes mellitus; prior heart failure hospitalization; actual study treatment; Grouped Geographical Region; composite of NT-proBNP or BNP *Z*-score; sex; age (years) and LVEF per 5

**Table 4:** Relation to outcome in cox regression interaction analysis

Univariable		ACM* 180 days	CVD 180 days	WHF 5 days	HF/RF Hosp 180 days	CVD + HF/RF Hosp 180 days
		HR (95% CI)				
IRF	Placebo	1.48 (1.15–1.91), *p* = 0.003	1.55 (1.17–2.07), *p* = 0.003	0.99 (0.68–1.45), *p* = 0.97	0.97 (0.76–1.22), *p* = 0.78	1.08 (0.88–1.31), *p* = 0.46
IRF	Treatment	0.92 (0.73–1.18), *p* = 0.53	0.98 (0.75–1.29), *p* = 0.91	0.75 (0.54–1.04), *p* = 0.08	0.90 (0.75–1.09), *p* = 0.29	0.93 (0.79–1.09), *p* = 0.37
Age/sex						
IRF	Placebo	1.62 (1.26–2.09), *p* = 0.0002	1.67 (1.26–2.23), *p* = 0.0005	1.02 (0.7–1.49), *p* = 0.90	0.98 (0.78–1.24), *p* = 0.87	1.11 (0.91–1.35), *p* = 0.31
IRF	Treatment	0.97 (0.76–1.24), *p* = 0.81	1.02 (0.78–1.35), *p* = 0.86	0.76 (0.55–1.06), *p* = 0.11	0.91 (0.75–1.1), *p* = 0.32	0.94 (0.8–1.11), *p* = 0.46
Full model^a^						
IRF	Placebo	1.35 (0.98–1.85), *p* = 0.07	1.47 (1.04–2.09), *p* = 0.03	0.84 (0.53–1.31), *p* = 0.44	0.86 (0.65–1.14), *p* = 0.31	0.96 (0.75–1.22), *p* = 0.72
IRF	Treatment	0.75 (0.54–1.04), *p* = 0.09	0.84 (0.59–1.19), *p* = 0.32	0.82 (0.54–1.24), *p* = 0.34	0.87 (0.69–1.1), *p* = 0.26	0.87 (0.71–1.07), *p* = 0.19

^a^WHF, REHOHFRF, COMP180: creatinine (µmol/L); hemoglobin (g/L); sodium (mmol/L); blood urea nitrogen (mg/dL); cerebrovascular accident; depression; asthma/bronchitis/COPD; atrial fibrillation/flutter; peripheral arterial occlusive disease; pulse (beats/min); respiration rate (breaths/min); systolic blood pressure (mmHg); edema; IV loop diuretics (total dose in furosemide units) at baseline; known history of diabetes mellitus; prior heart failure hospitalization; actual study treatment; Grouped Geographical Region; composite of NT-proBNP or BNP *Z*-score; sex; age (years) and LVEF per 5. CVD/ACM: creatinine (µmol/L); hemoglobin (g/L); sodium (mmol/L); blood urea nitrogen (mg/dL); asthma/bronchitis/COPD; peripheral arterial occlusive disease; respiration rate (breaths/min); systolic blood pressure (mmHg); body mass index (kg/m^2^); edema; IV loop diuretics (total dose in furosemide units) at baseline; known history of diabetes mellitus; prior heart failure hospitalization; actual study treatment; composite of NT-proBNP or BNP *Z*-score; sex; age (years) and LVEF per 5

Inversely, in adjusted models in the overall RELAX-AHF2 population, worsening renal function was associated with increased risk of all-cause mortality [HR 1.39 (95% CI 1.06–1.82), *p* = 0.02], worsening HF [HR 1.97 (95% CI 1.46–2.67), *p* < 0.0001], and the composite endpoint of CV death and/or hospitalization for HF or renal failure [HR 1.24 (95% CI 1.03–1.49), *p* = 0.02], Table 3). With regard to worsening renal function, no treatment interaction was observed regarding all outcomes (all-cause mortality *p*-interaction = 0.51, CV death *p*-interaction = 0.15, worsening HF *p*-interaction = 0.69, hospitalization for HF/renal failure *p*-interaction = 0.45, composite of CV death and/or hospitalization for HF/renal failure *p*-interaction = 0.10, Table 3).

For both improvement in renal function and worsening renal function, no association with outcome was observed, and no treatment interaction was found in the sensitivity analyses performed in the RELAX-AHF cohort (Supplementary Table 5).

### Renal function, treatment effect and outcome in mediation analysis

In RELAX-AHF2, improvement in renal function between baseline and day 2 as binary mediator (Indirect effect) did not mediate the treatment effect (Direct effect) of serelaxin regarding the outcomes cardiovascular death, all-cause mortality, or the combined endpoint hospitalization for heart failure or renal failure [indirect effect of cardiovascular death HR 1.01 (0.99–1.04), indirect effect of all-cause mortality HR 1.01 (0.99–1.03), indirect effect of HF/RF hospitalization HR 0.99 (0.97–1.00), Table 5, Central illustration]. Similar results were found in the sensitivity analyses performed in the RELAX-AHF cohort (Supplementary Table 6).

**Table 5:** Mediation analysis

RELAX-AHF-2	CV death	All-cause mortality	HF/renal failure hospitalization
	Hazard ratio (95% CI)		
Total effect	0.95 (0.79, 1.12)	0.91 (0.77, 1.04)	0.95 (0.85, 1.08)
Direct effect (treatment)	0.93 (0.78, 1.11)	0.90 (0.76, 1.04)	0.97 (0.86, 1.09)
Indirect effect (renal function)	1.01 (0.99, 1.04)	1.01 (0.99, 1.03)	0.99 (0.97, 1.00)

## Discussion

In this analysis in patients with acute heart failure included in the RELAX-AHF trials, serelaxin treatment was associated with improvement in renal function between baseline and day 2. However, any potential treatment effect of serelaxin on CV death and all-cause mortality was not mediated by improvement in renal function between baseline and day 2.

### Effect of serelaxin on renal function in heart failure

Serelaxin, the recombinant form of human relaxin, was shown to be safe in patients with acute heart failure and mild-to-moderate renal insufficiency in the pre-RELAX-AHF, RELAX-AHF and RELAX-AHF2 trials [9]. Confirming the proposed beneficial renal effects, serelaxin was found to improve renal function in both the RELAX-AHF and the successive RELAX-AHF2 trial. In RELAX-AHF, serelaxin treatment was furthermore associated with less worsening of markers of renal function such as cystatin-C, creatinine, blood urea nitrogen and uric acid concentration at day 2 [6, 7, 13]. Again, this was confirmed in the post hoc renal substudy, where serelaxin was associated with significantly lower serum creatinine and plasma cystatin-C values in the first 5 days and with lower incidence of worsening renal function at day 2 [7]. The proposed mechanism responsible for the observed improvement in renal function associated with serelaxin treatment is an increase in renal blood flow [14, 15]. This is thought to be a result of decreased systemic vascular resistance, caused by the binding of serelaxin to receptors on endothelial cells of the vasculature, activating the vascular endothelin B receptor, vascular endothelial growth factor and nitric oxide production [16].

It was frequently suggested that the improvement in renal function partly explained the beneficial effects of serelaxin on CV death in RELAX-AHF [9]. After observing a significant improvement in renal function with serelaxin in the pre-RELAX, RELAX-AHF and RELAX-AHF2 trials, it was hypothesized that improvement in renal function in patients with acute heart failure might favorably affect cardiovascular event rates and that perhaps any potential beneficial effect of serelaxin might be more pronounced in—or even limited to—patients experiencing improvement in renal function with serelaxin. Therefore, despite the neutral findings of the RELAX-AHF2 trial on clinical outcomes, the beneficial effects of relaxin on renal function in patients with acute heart failure could identify a subgroup of patients that will experience benefit from relaxin treatment.

Hence, we aimed to study whether those patients with a clear improvement in renal function on serelaxin might have achieved benefit in terms of improving CV outcomes. This study shows that the consistent benefit seen with serelaxin on improvement in renal function does not translate into any potential improvement in cardiovascular outcomes. Moreover, our presented mediation analyses show that any potential beneficial effect of serelaxin on (cardiovascular) mortality could not be explained through improvement in renal function between baseline and day 2. Current analyses showed no indirect effect of improvement in renal function between baseline and day 2 on treatment efficacy, regarding cardiovascular death, all-cause mortality, and the combined endpoint of hospitalization for heart failure or renal failure.

### Changes in renal function in acute heart failure and associated outcome

Another interesting finding of the present study was that improvement in renal function in patients allocated to placebo was associated with increased risk of all-cause mortality and cardiovascular death at 180 days, which remained consistent for cardiovascular death after multivariable adjustment. These associations were not observed in patients with improvement in renal function receiving serelaxin. Furthermore, in this current study, worsening renal function was associated with increased risk of all-cause mortality at 180 days, worsening heart failure at 5 days, and the composite endpoint of cardiovascular death or hospitalization for heart failure or renal failure at 180 days. No treatment interaction with worsening renal function was observed.

The prognostic implications of change in renal function, either an improvement or worsening of renal function, on clinical outcome in acute heart failure have been well established [1, 4, 5, 9, 17, 18]. Testani et al. describe improvement in renal function in patients hospitalized for acutely decompensated heart failure as a frequently observed event, which is associated with increased risk for subsequent mortality [4]. Patients who experience improvement in renal function frequently have a high incidence of pre-admission worsening renal function and therefore high serum creatinine levels at admission, which makes them more prone to experience improvement in renal function [4, 5]. Therefore, excess risk of pre-admission renal dysfunction might be attributed to improvement in renal function.

Perhaps not the occurrence but rather the underlying cause of improvement in or worsening of renal function is the reason for concern. In acute heart failure, the cause of changes in creatinine is often indirect and might, for example, represent a functional decline in eGFR without the presence of renal injury [19]. Moreover, if worsening renal function occurs in the context of a favorable diuretic response, it is not associated with poor outcome [18, 20]. The clinical context in which changes in creatinine occur, such as a favorable diuretic response, is of great importance, as these changes then can be dubbed pseudo-worsening renal function and in this circumstance are not associated with worse outcome. [21]

In previous studies, serelaxin was not associated with better diuretic response [22], so therefore, one can argue that the improvement in renal function associated with serelaxin treatment might not be caused by resolution of venous congestion, but perhaps due to direct effects on the kidney. In our study, increase in hematocrit was found to be an independent predictor of improvement in renal function, supporting the hypothesis that improvement in renal function observed with serelaxin might not be due to decongestion. Moreover, this might explain why improvement in renal function associated with serelaxin was not associated with poor outcomes in our study as additional mechanisms may be involved.

### Cardiorenal endpoints in heart failure trials

The present study implicates that the change in renal function might not be a suitable surrogate outcome parameter in phase-2 trials to support the design and execution of phase 3 trials. Even more general, it might be argued whether therapeutics that improve renal function in heart failure also improve clinical outcomes. For instance, the Placebo-controlled Randomized Study of Selective A1 Adenosine Receptor Antagonist Rolofylline for Patients Hospitalized with Acute Decompensated Heart Failure and Volume Overload to Assess Treatment Effect on Congestion and Renal Function (PROTECT) study was designed to investigate the hypothesis that improvement in renal function with rolofylline might lead to subsequent improvement in clinical outcomes in patients with acute HF [23, 24]. The rationale behind the study design was the positive data of the phase-II trials evaluating rolofylline, an adenosine A1-receptor antagonist, where treatment in addition to loop diuretics was found to prevent increases in serum creatinine and enhanced diuresis when compared to placebo [25, 26]. However, despite the results of the phase 1 and 2 studies, the PROTECT study failed to show benefit of rolofylline over placebo on outcomes, as well in reducing worsening renal function.

Results from the Diuretic Optimization Strategies Evaluation (DOSE) trial, where a linear increase in creatinine was associated with improved outcomes, also argue against using serum creatinine changes as a surrogate endpoint in trials with decongestive therapies [27]. Also, data from the Cardiorenal Rescue Study in Acute Decompensated Heart Failure (CARRESS-HF) study suggest that change in creatinine might not be a suitable endpoint in trials with patients admitted for acute HF [28]. Our data are in line with these studies and contribute to the understanding of cardiorenal interactions in heart failure.

## Future perspectives

To our knowledge, this is the first analysis evaluating whether any potential beneficial effects of serelaxin treatment on cardiovascular outcome might be limited to or more pronounced in patients experiencing improvement in renal function. Despite the paucity of data with regard to the underlying mechanism of changes in renal function and (contrasting) corresponding outcomes in acute heart failure, there seems to be a bidirectional relationship between change in renal function and cardiac function. The hypothesis that improvement in renal function might be the driving factor of serelaxin efficiency in reducing cardiovascular outcomes has not been confirmed by our results. However, data on renal hemodynamics and (se)relaxin treatment in patients with acute heart failure are lacking and should be further investigated. Upcoming future trials investigating relaxin in patients with acute heart failure should perhaps reconsider using improvement in renal function as a surrogate marker for cardiovascular efficacy.

### Limitations

As this is a post hoc and subgroup analysis of two clinical trials, results should be considered hypothesis-generating and not necessarily applicable to the general acute HF population. The RELAX-AHF2 trial was neutral, and therefore, identification of subgroups remains difficult. Change of type 1 errors cannot be ruled out, given that the study was conducted in a subset of the population (patients with creatinine values available at baseline and day 2). In the RELAX-AHF study, patients with an eGFR < 30 mL/min/1.73 m^2^ and in the RELAX-AHF2 patients with an eGFR < 25 mL/min/1.73 m^2^ were excluded and inclusion of this group could have altered the results, as serum creatinine at baseline was a predictor of improvement in renal function in this analysis. Future research should be performed to confirm our study results.

## Conclusions

Serelaxin improved renal function in two large trials with patients who were admitted for acute heart failure. However, any potential effect of serelaxin on cardiovascular mortality was not mediated by an improvement in renal function between baseline and day 2. These data suggest that improvement in renal function might not be a suitable surrogate marker for potential efficacy in future trials with novel relaxin agents in acute heart failure.

## Clinical perspectives

In phase-2 trials, improvement in renal function is often considered a surrogate endpoint for efficacy in potential phase 3 trials, as it is generally assumed that drugs that improve renal function might have beneficial effects on mortality and heart failure hospitalizations. However, results from our study suggest that any potential effects of serelaxin on clinical outcome were not mediated by improvement in renal function. These data increase our understanding of cardiorenal interactions in heart failure and might provide guidance on the design of clinical development programs for upcoming novel relaxin agents.

## Supplementary Information

Below is the link to the electronic supplementary material.

Supplementary file1 (DOCX 49 kb)
