# Supplementary material for: Disconnect between the effects of serelaxin on renal function and outcome in acute heart failure
Source: Clin Res Cardiol. 2023 Jan 19;112(7):901–10. doi: 10.1007/s00392-022-02144-6 (PMC10293419; doi:10.1007/s00392-022-02144-6)
Supplement: Supplementary file 1 — Supplementary file1 (DOCX 49 kb) [file 392_2022_2144_MOESM1_ESM.docx]

**Supplemental Material**

to

**Disconnect between the effects of serelaxin on renal function and outcome in acute heart failure**

I. E. Beldhuis^a^, J.M. ter Maaten^a^, S. Figarska^a^, K. Damman^a^, P.S. Pang^b^, B. Greenberg^c^, B.A. Davison^d^, G. Cotter^d^, T. Severin^e^, C. Gimpelewicz^e^, G. M. Felker^f^, G. Filippatos^g^, J. R. Teerlink^h^, M. Metra^i^, A. A. Voors^a^.

**Supplementary Table 1. Baseline characteristics by treatment for patients who did and did not experience improvement in renal function between baseline and day 2 in RELAX-AHF2**

**Supplementary Table 2. Baseline characteristics for patients who did and did not experience improvement in renal function between baseline and day 2 in RELAX-AHF**

**Supplementary Table 3. Baseline characteristics by treatment for patients who did and did not experience improvement in renal function between baseline and day 2 in RELAX-AHF**

**Supplementary Table 4. Occurrence of improvement in renal function and worsening renal function in RELAX-AHF**

**Supplementary Table 5. Improvement in renal function/worsening renal function and relation to outcome in cox regression analysis in RELAX-AHF**

**Supplementary Table 6. Mediation analysis in validation cohort (RELAX-AHF)**

**Supplementary Table 1. Baseline characteristics by treatment for patients who did and did not experience improvement in renal function between baseline and day 2 in RELAX-AHF2**

| **RELAX-AHF-2** | **Placebo** | | **Serelaxin** | | **P-value** |
| --- | --- | --- | --- | --- | --- |
|  | **IRF** | **No IRF** | **IRF** | **No IRF** |  |
| **N=6285** | **405**  **(13%)** | **2743**  **(87%)** | **681**  **(22%)** | **2456**  **(78%)** |  |
| **Demographics** |  |  |  |  |  |
| Age, yrs | 70 ± 12 | 73 ± 11) | 71 ± 12 | 74 ± 11 | **<0.001** |
| Female | 163 (40) | 1125 (41) | 244 (36) | 996 (41) | 0.10 |
| White race | 382 (94) | 2517 (92) | 621 (91) | 2278 (93) |  |
| Body mass index, kg/m2 | 29.9 ± 6.6 | 29.8 ± 6.3) | 30.2 ± 6.5 | 39.7 ± 6.3 | 0.40 |
| LVEF, % | 39 ± 14 | 40 ± 14) | 38 ± 14 | 41 ± 14 | 0.07 |
| HFrEF | 217 (56) | 1343 (52) | 370 (58) | 1141 (49) | **<0.001** |
| NYHA functional class |  |  |  |  | 0.69 |
| I | 10 (3) | 81 (4) | 23 (5) | 88 (5.0) |  |
| II | 126 (41) | 778 (39) | 188 (38) | 694 (39) |  |
| III | 134 (43) | 921 (46) | 231 (46) | 813 (46) |  |
| IV | 40 (13) | 211 (11) | 60 (12) | 180 (10) |  |
| Previous HFH | 227 (59) | 1403 (55) | 344 (54) | 1245 (54) | 0.29 |
| Ischemic etiology | 170 (54) | 1083 (53) | 264 (52) | 1000 (56) | 0.37 |
| Systolic blood pressure, mmHg | 139 ± 13 | 142 ± 15) | 140 ± 14 | 142 ± 16 | **<0.001** |
| Heart rate, beats/min | 81 ± 17 | 82 ± 16) | 83 ± 17 | 81 ± 16 | 0.06 |
| **Biomarkers baseline** |  |  |  |  |  |
| Creatinine, mg/dL | 1.48 ± 0.38 | 1.34 ± 0.39) | 1.43 ± 0.35 | 1.34 ± 0.38 | **<0.001** |
| Baseline eGFR CKD-EPI | 45.8 ± 14.0 | 51.1 ± 15.2) | 47.8 ± 13.7 | 50.9 ± 15.1 | **<0.001** |
| Day 2 eGFR CKD-EPI | 64.6 ± 19.4 | 48.1 ± 16.4) | 68.4 ± 18.8 | 49.8 ± 17.2 | **<0.001** |
| Potassium, mmol/L | 4.4 ± 0.6 | 4.3 ± 0.6) | 4.3 ± 0.6 | 4.3 ± 0.6 | **0.04** |
| Hemoglobin, g/L | 127 ± 20.2 | 126 ± 19.5) | 129 ± 19.7 | 126 ± 19.5 | **0.002** |
| BUN, mg/dL | 26.6 [20.1; 36.9] | 23.8 [18.2; 31.7] | 25.0 [19.3; 33.1] | 24.0 [18.8; 31.7] | **<0.001** |
| AST, IU/L | 29.3 [21.0; 41.9] | 26.0 [20.0; 36.0] | 29.2 [21.5; 43.0] | 25.0 [19.0; 35.0] | **<0.001** |
| NT-proBNP, ng/L | 4695 [2825; 9637] | 5101 [2956; 9499] | 5785 [2973; 8948] | 5380 [3022; 10066] | 0.91 |
| **Medical therapy (baseline (n(%))** |  |  |  |  |  |
| ACEi/ARB | 277 (72) | 1825 (70) | 432 (68) | 1585 (68) | 0.12 |
| Beta-blocker | 298 (78) | 1942 (75) | 492 (78) | 1742 (74) | 0.18 |
| MRA | 141 (37) | 792 (31) | 208 (33) | 667 (29) | **0.004** |

*Values are mean ± SD, frequency (percentage), or median (interquartile range). ACEi= angiotensin converting enzyme inhibitor, ARB=angiotensin receptor blocker, AST=aspartate aminotransferase, BUN= blood urea nitrogen, CKD-EPI=chronic kidney disease epidemiology collaboration, eGFR=estimated glomerular filtration rate, HFH= heart failure hospitalization, IRF = improvement in renal function, LVEF=left ventricular ejection fraction, MRA=mineralocorticoid receptor antagonist, NT-proBNP=N-terminal prohormone brain natriuretic peptide, NYHA=New York Heart Association.*

**Supplementary Table 2. Baseline characteristics for patients who did and did not experience improvement in renal function between baseline and day 2 in RELAX-AHF**

| **RELAX-AHF**  N=1090 | **No IRF** | **IRF** | **P-value** |
| --- | --- | --- | --- |
|  | **N=951**  **(87%)** | **N=139**  **(13%)** |  |
| **Demographics** |  |  |  |
| Age, yrs | 73 (11) | 69.6 (12) | **0.005** |
| Female | 355 (37) | 59 (42) | 0.29 |
| White race | 906 (95) | 133 (96) | 0.99 |
| Body mass index, kg/m2 | 29 (6) | 30.0 (6) | 0.15 |
| LVEF, % | 39 (14) | 38.2 (16) | 0.80 |
| NYHA functional class |  |  | 0.06 |
| I | 19 (3) | 1 (1) |  |
| II | 240 (35) | 45 (40) |  |
| III | 302 (45) | 58 (51) |  |
| IV | 118 (17) | 10 (9) |  |
| Congestive HF history | 689 (73) | 114 (82) | **0.02** |
| Ischemic heart disease history | 500 (53) | 69 (50) | 0.58 |
| Diabetes mellitus status | 455 (48) | 65 (47) | 0.88 |
| Atrial fibrillation/flutter history | 482 (51) | 84 (60) | **0.04** |
| Systolic blood pressure, mmHg | 143 (16) | 140 (16) | **0.03** |
| **Biomarkers** |  |  |  |
| Creatinine, mg/dL | 1.30 (0.37) | 1.40 (0.38) | **0.007** |
| Baseline eGFR CKD-EPI, ml/min/1.73m2 | 53.7 (16.7) | 49.1 (13.7) | **<0.001** |
| Day 2 eGFR CKD-EPI, ml/min/1.73m2 | 51.7 (18.1) | 69.0 (18.4) | **<0.001** |
| NT-proBNP, ng/L | 3000 [2697;5517] | 3000 [2546;7458] | 0.21 |
| **Medical therapy** |  |  |  |
| ACEi/ARB | 657 (69) | 88 (63) | 0.20 |
| Beta-blocker | 148 (16) | 30 (22) | 0.10 |
| **Treatment** |  |  | **<0.001** |
| Placebo | 504 (53) | 43 (31) |  |
| Serelaxin, 30ug/kg/day | 447 (47) | 96 (69) |  |

*Values are mean ± SD, frequency (percentage), or median (interquartile range). ACEi= angiotensin converting enzyme inhibitor, ARB=angiotensin receptor blocker, CKD-EPI=chronic kidney disease epidemiology collaboration, eGFR=estimated glomerular filtration rate, IRF = improvement in renal function, LVEF=left ventricular ejection fraction, NT-proBNP=N-terminal prohormone brain natriuretic peptide, NYHA=New York Heart Association.*

**Supplementary Table 3. Baseline characteristics** **by treatment for patients who did and did not experience improvement in renal function between baseline and day 2 in RELAX-AHF**

| **RELAX-AHF**  N=1090 | **Placebo** | | **Serelaxin** | | **P-value** |
| --- | --- | --- | --- | --- | --- |
|  | **IRF** | **No IRF** | **IRF** | **No IRF** |  |
| **N=1090** | **43**  **(8%)** | **504**  **(92%)** | **96**  **(18%)** | **447**  **(72%)** |  |
| **Demographics** |  |  |  |  |  |
| Age, yrs | 72 (11) | 73 (11) | 69 (12) | 72 (11) | **0.013** |
| Female | 22 (51) | 191 (38) | 37 (39) | 164 (37) | 0.32 |
| White race | 41 (95) | 483 (96) | 92 (96) | 423 (95) | 0.85 |
| Body mass index, kg/m2 | 29 (7) | 29 (6) | 30 (6) | 29 (5) | 0.10 |
| LVEF, % | 38 (14) | 38 (14) | 38 (16) | 39 (14) | 0.95 |
| NYHA functional class |  |  |  |  |  |
| I | 0 (0) | 9 (3) | 1 (1) | 10 (3) |  |
| II | 17 (50) | 113 (31) | 28 (35) | 127 (40) |  |
| III | 13 (38) | 174 (48) | 45 (56) | 128 (40) |  |
| IV | 4 (12) | 65 (18) | 6 (8) | 53 (17) |  |
| Congestive HF history | 34 (79) | 364 (72) | 80 (83) | 325 (73) | 0.11 |
| Ischemic heart disease history | 17 (40) | 275 (55) | 52 (54) | 225 (50) | 0.20 |
| Diabetes mellitus status | 20 (47) | 241 (48) | 45 (47) | 214 (48) | 1.00 |
| Atrial fibrillation/flutter history | 28 (65) | 260 (52) | 56 (58) | 222 (50) | 0.14 |
| Systolic blood pressure, mmHg | 137 (14) | 143 (17) | 141 (16) | 143 (16) | 0.10 |
| **Biomarkers** |  |  |  |  |  |
| Creatinine, mg/dL | 1.41 (0.41) | 1.30 (0.38) | 1.39 (0.37) | 1.30 (0.37) | **0.05** |
| Baseline eGFR CKD-EPI | 46.8 (12.4) | 53.5 (16.7) | 50.1 (14.2) | 54.0 (16.8) | **0.01** |
| Day 2 eGFR CKD-EPI | 64.5 (17.4) | 49.9 (17.3) | 71.0 (18.5) | 53.7 (18.8) | **<0.001** |
| NT-proBNP, ng/L | 3426 [3000;6146] | 3000 [2734;5805] | 3000 [2405;7684] | 3000 [2674;5197] | 0.52 |
| **Medical therapy** |  |  |  |  |  |
| ACEi/ARB | 31 (72) | 347 (69) | 57 (59) | 310 (69) | 0.25 |
| Beta-blocker | 7 (16) | 92 (18) | 23 (24) | 56 (13) | **0.02** |

*Values are mean ± SD, frequency (percentage), or median (interquartile range). ACEi= angiotensin converting enzyme inhibitor, ARB=angiotensin receptor blocker, CKD-EPI=chronic kidney disease epidemiology collaboration, eGFR=estimated glomerular filtration rate, IRF = improvement in renal function, LVEF=left ventricular ejection fraction, NT-proBNP=N-terminal prohormone brain natriuretic peptide, NYHA=New York Heart Association.*

**Supplementary Table 4. Occurrence of improvement in renal function and worsening renal function in RELAX-AHF**

| **RELAX-AHF** | **Total population**  **(N(%))** | **Placebo (N(%))** | **Serelaxin (N(%))** | **Odds ratio (95% CI)** | **P-value** |
| --- | --- | --- | --- | --- | --- |
| **IRF** | 139 (13%) | 43 (8%) | 96 (18%) | 2.52 (1.72-3.69) | <0.0001 |
| **WRF** | 124 (11%) | 78 (14%) | 46 (8%) | 0.56 (0.38-0.82) | 0.003 |

**Supplementary Table 5. Improvement in renal function/worsening renal function and relation to outcome in cox regression analysis in RELAX-AHF**

| **RELAX-AHF** | **All-cause mortality**  **180 days** | **CV Death**  **180 days** | **CVD + HF/RF Hosp**  **60 days** |
| --- | --- | --- | --- |
| **Univariable** | **Hazard Ratio (95% CI)** | | |
| **IRF** | 1.44 (0.85-2.43), p=0.17 | 1.33 (0.73-2.41), p=0.35 | 0.89 (0.53-1.50), p=0.66 |
| **WRF** | 1.33 (0.76-2.35), p=0.32 | 1.70 (0.95-3.02), p=0.07 | 1.33 (0.83-2.13), p=0.24 |
| **Age/sex** |  |  |  |
| **IRF** | 1.56 (0.92-2.64), p=0.10 | 1.42 (0.78-2.58), p=0.25 | 0.92 (0.55-1.55), p=0.76 |
| **WRF** | 1.33 (0.76-2.35), p=0.32 | 1.70 (0.95-3.03), p=0.07 | 1.33 (0.83-2.13), p=0.24 |
| **Full model*** |  |  |  |
| **IRF** | 0.98 (0.43-2.25), p=0.96 | 0.54 (0.18-1.59), p=0.26 | 1.24 (0.6-2.53), p=0.56 |
| **WRF** | 1.46 (0.6-3.54), p=0.40 | 1.84 (0.74-4.57), p=0.19 | 0.97 (0.37-2.52), p=0.95 |
| **Treatment interaction** |  |  |  |
| **IRF** | 0.89 | 0.75 | 0.28 |
| **WRF** | 0.52 | 0.49 | 0.33 |

**** All-cause mortality 180 days;*** *Age (yrs), CHF 1 month previously, stroke or other cerebrovascular event, respiratory rate (1 breath/min), systolic blood pressure (10mmHg), Edema (2/3 vs 0/1), Orthopnea (2/3 vs 0/1), lymphocyte %, sodium (mmol), creatinine (mg/dL), log2 troponin T.*

***CV death 180 days;*** *USA-like region, systolic blood pressure, orthopnea, angina, hyperthyroid, mitral regurgitation, atrial fibrillation or flutter at screening, white blood cell count, lymphocyte %, blood urea nitrogen, sodium, potassium, calcium, total protein, troponin, and NT-proBNP.*

***CV death or HF/RF rehospitalization 60 days:*** *NYHA class 30 days prior; systolic blood pressure; respiratory rate; number of HF hospitalizations in past year; orthopnoea; asthma, bronchitis, or COPD; hyperthyroid; lymphocyte %; blood urea nitrogen; phosphate; sodium; and total protein.*

**Supplementary Table 6. Mediation analysis in validation cohort (RELAX-AHF)**

| **RELAX-AHF** | **CV Death** | **All-cause mortality** |
| --- | --- | --- |
|  | **Hazard Ratio (95% CI)** | |
| **Total effect** | 0.63 (0.40, 0.96) | 0.65 (0.44, 0.94) |
| **Direct effect (treatment)** | 0.63 (0.39, 0.96) | 0.64 (0.43, 0.94) |
| **Indirect effect (renal function)** | 1.01 (0.99, 1.02) | 1.01 (1.00, 1.02) |
